# Lipid Mediators Derived from Docosahexaenoic Acid Alleviate Periodontal Inflammation and Alveolar Bone Loss in Periodontitis Associated with Suppression of the NF-κB and STAT3 Signaling Pathways

**DOI:** 10.3390/nu18162659

**Published:** 2026-08-14

**Authors:** Yan Su, Soon Kyu Kwon, Hack Sun Choi, Yunjon Han, Yong-Suk Jang, Jung-Hee Park, Jong Hyun Choi, Jeong-Woo Seo

**Affiliations:** 1 Microbial Biotechnology Research Center, Korea Research Institute of Bioscience and Biotechnology (KRIBB), Jeongeup 56212, Republic of Korea; suyan@kribb.re.kr (Y.S.); ksk0469@kribb.re.kr (S.K.K.); gugin@kribb.re.kr (Y.H.); jhchoi@kribb.re.kr (J.H.C.); 2 Department of Biotechnology, Jeonbuk National University, Iksan 54596, Republic of Korea; junghee.park@jbnu.ac.kr; 3 Department of Biochemistry & Molecular Biology, Yonsei University College of Medicine, Seoul 03722, Republic of Korea; choix074@yuhs.ac; 4 Department of Molecular Biology, Jeonbuk National University, Jeonju 54896, Republic of Korea; yongsuk@jbnu.ac.kr; 5 Department of Bioactive Material Sciences, Jeonbuk National University, Jeonju 54896, Republic of Korea

**Keywords:** lipid mediators, periodontitis, gingival fibroblasts, alveolar bone loss, NF-κB, STAT3

## Abstract

**Background**: Lipid mediators (LM), comprising 17S-hydroxy-docosahexaenoic acid, resolvin D5, and protectin DX in a ratio of 3:47:50, were naturally generated from docosahexaenoic acid by soybean lipoxygenase and exhibit potent anti-inflammatory activities. However, their therapeutic potential in periodontitis (PD) remains unclear. Therefore, this study investigated the protective effects of LM against PD in vitro and in vivo. **Methods**: Human gingival fibroblasts (HGFs) were stimulated with *Porphyromonas gingivalis* lipopolysaccharide (PG-LPS) to evaluate the anti-inflammatory effects of LM. Nitric oxide (NO) production, prostaglandin E2 (PGE2) level, inflammatory cytokines secretion, matrix metalloproteinase 9 (MMP9) expression, and NF-κB/STAT3 signaling pathways were analyzed. In vivo, a ligature-induced rat model of PD was established, and LM were orally administered at doses of 5, 10, and 20 μg/kg once daily. The effects of LM were evaluated by histopathological examination, micro-computed tomography (micro-CT), quantitative real-time PCR (qRT-PCR), and immunohistochemistry (IHC). **Results**: In PG-LPS-stimulated HGFs, LM significantly reduced NO and PGE2 production by downregulating inducible nitric oxide synthase and cyclooxygenase-2 expression. Moreover, LM decreased the levels of interleukin (IL)-6, tumor necrosis factor-α (TNF-α), IL-1β, and MMP9, accompanied by suppression of the NF-κB and STAT3 signaling pathways. In the ligature-induced rat model of PD, LM significantly attenuated gingival inflammation and alveolar bone loss. Furthermore, IHC analysis revealed that LM markedly reduced the level of IL-6, TNF-α, MMP9, and phosphorylated STAT3 in gingival tissues. **Conclusions**: LM exerted protective effects against periodontitis by alleviating inflammatory response and reducing alveolar bone loss, which may be associated with suppression of the NF-κB and STAT3 signaling pathways.

## 1. Introduction

Periodontitis (PD) is a chronic inflammatory disease that progressively destroys the tissues and alveolar bone, ultimately compromising oral health [1,2]. Early-stage PD is characterized by gingival redness, swelling, and bleeding, which are hallmarks of gingival tissue damage. Such damage compromises the protective function of the gingiva and disrupts periodontal homeostasis [3,4]. As the disease progresses, persistent inflammation promotes extracellular matrix degradation and alveolar bone resorption, ultimately resulting in tooth loss [5,6].

The primary drivers of PD are pathogenic microbial communities that trigger excessive production of pro-inflammatory cytokines and matrix metalloproteinases (MMPs), ultimately leading to periodontal tissue destruction [7]. Among these bacterial factors, *Porphyromonas gingivalis* lipopolysaccharide (PG-LPS) plays a crucial role in disease progression [8,9]. Gingival fibroblasts are the predominant cells of gingival connective tissue and play a central role in maintaining tissue homeostasis as well as mediating periodontal inflammation [10]. Upon PG-LPS stimulation, gingival fibroblasts participate in an innate immune response by activating inflammatory signaling pathways, including the NF-κB and STAT3 pathways. NF-κB rapidly induces the transcription of pro-inflammatory mediators, whereas STAT3 amplifies inflammatory responses and promotes osteoclastogenesis. Consequently, the production of pro-inflammatory mediators, including tumor necrosis factor-α (TNF-α), interleukin (IL)-1β, IL-6, prostaglandin E2 (PGE2), and matrix metalloproteinase 9 (MMP9) is markedly upregulated, thereby promoting extracellular matrix degradation and periodontal tissue destruction [11,12,13,14].

Despite advances in the clinical management of periodontitis, current therapeutic strategies, including mechanical debridement, antibiotics, and anti-inflammatory drugs, primarily focus on reducing the bacterial burden and suppressing inflammation rather than promoting the resolution of inflammation and restoring periodontal homeostasis [15]. Specialized pro-resolving mediators (SPMs), first characterized by Serhan, are a family of endogenous lipid mediators that include resolvins, lipoxins, protectins, and maresins [16]. Unlike conventional anti-inflammatory agents that primarily suppress immune responses, SPMs promote the resolution of inflammation by limiting neutrophil infiltration, enhancing macrophage-mediated clearance of inflammatory cells and debris, and promoting tissue repair and regeneration [17]. Resolvins have demonstrated considerable therapeutic potential for the treatment of PD [18]. For example, resolvin E1 (RvE1) has been shown to reduce inflammatory cell infiltration, prevent alveolar bone loss, and promote periodontal tissue regeneration [19]. Similarly, RvD1 protected periodontal fibroblasts by reversing IL-1β-induced inflammatory responses, restoring cell proliferation and wound-healing capacity, and re-establishing extracellular matrix homeostasis [20]. Despite these promising findings, most studies have focused on chemically synthesized individual SPMs, whereas the therapeutic potential of naturally generated DHA-derived lipid mediators mixtures remains poorly understood.

The lipid mediators (LM) were naturally generated from docosahexaenoic acid (DHA) using soybean lipoxygenase in our laboratory. HPLC and LC-MS/MS analyses confirmed the LM consisted primarily of 17S-hydroxydocosahexaenoic acid (17S-HDHA), resolvin D5 (RvD5), and protectin DX (PDX), with an approximate relative composition of 3:47:50. Detailed procedures for LM preparation and chemical characterization were provided in the Supplementary file. In our previous studies, LM exhibited protective effects in inflammatory diseases, including atopic dermatitis and arthritis [21,22,23]. However, the therapeutic potential in periodontitis remains unclear. Therefore, in the present study, we evaluated the protective effects of LM against PD using human gingival fibroblasts (HGFs) and a ligature-induced rat model of periodontitis.

## 2. Materials and Methods

### 2.1. Animals

Male Sprague-Dawley rats (7 weeks old) were purchased from Orient Bio (Gyeonggi, Republic of Korea). The animals were housed under controlled conditions at 21–23 °C, with 60–70% relative humidity and a 12 h light/dark cycle. Before the experiments, all rats were allowed to acclimate to the new environment. This study was approved by the Institutional Animal Care and Use Committee of the Korea Research Institute of Bioscience & Biotechnology (Daejeon, Republic of Korea) and the Institutional Animal Ethics Committee (KRIBB-AEC-23335).

### 2.2. Ligature-Induced PD Rat Model

The ligature-induced periodontitis model is one of the most widely used and well-established experimental models because the placement of the ligature promotes rapid accumulation of endogenous oral bacteria, resulting in robust inflammatory responses and alveolar bone loss that resemble key pathological features of human periodontitis. A rat PD model of periodontitis was established using 3-0 silk ligatures, as previously described [24,25]. Briefly, the rats were anesthetized using isoflurane, and ligatures were placed within the gingival sulcus around the interdental regions between the maxillary second and third molars to induce periodontitis. Ligature placement was performed under adequate general anesthesia to minimize procedural pain and distress. Animals were monitored until recovery from anesthesia and assessed daily for general health and signs of pain or distress, including changes in body weight, food/water intake, locomotor activity, posture, facial expression, mastication, and local oral conditions (gingival swelling, bleeding, or ulceration). Animals showing persistent or severe signs of pain or distress were humanely euthanized in accordance with institutional animal welfare guidelines. No animals required early euthanasia during the study.

The rats were randomly assigned to five groups as follows (*n* = 5/group): A negative control (NC) group treated with sterile saline; a PD model group induced by ligature placement and treated with sterile saline; and three treatment groups subjected to ligature placement and orally administered LM at doses of 5, 10, and 20 μg/kg once daily, starting on the day of ligature placement. LM were dissolved in ethanol to prepare a stock solution and then diluted with sterile saline before administration. The doses of LM were selected based on our previous study [23]. After 4 weeks, the rats were euthanized under anesthesia, the maxillae together with the surrounding gingival tissues were harvested for subsequent analyses. Gingival tissues from all animals were used for qRT-PCR analysis. For micro-computed tomography (micro-CT), histological (H&E), and immunohistochemical (IHC) analyses, three animals were randomly selected from each group.

The gingival tissues were fixed in 4% paraformaldehyde, embedded in paraffin, sliced into thin sections, and stained with hematoxylin–eosin (H&E). Alveolar bone morphology was evaluated by micro-CT using a SkyScan 1276 scanner (Bruker, Kontich, Belgium) at a resolution of 17.25 μm and a voltage of 70 kV [23]. Alveolar bone loss was quantified by measuring the distance between the cementoenamel junction (CEJ) and the alveolar bone crest (ABC) [24]. Bone volume fraction (BV/TV) and bone mineral density (BMD) were quantified using CTAn software 1.20.3.0 (Bruker). All histological assessments and micro-CT analysis were performed by investigators blinded to the experimental groups to minimize observer bias.

### 2.3. Immunohistochemistry Analysis

Paraffin-embedded gingival tissue sections were deparaffinized, treated with 3% hydrogen peroxide to block endogenous peroxidase activity, and subjected to antigen retrieval using citrate buffer. The sections were incubated overnight at 4 °C with primary antibodies (Abcam, Cambridge, UK) against IL-6 (ab9324, 1:1000), TNF-α (ab215188, 1:1000), MMP9 (ab58803, 1:100) and phosphorylated STAT3 (pSTAT3, ab32143, 1:200), followed by incubation with the corresponding secondary antibody (Abcam) for 2 h at 37 °C in the dark. Finally, the sections were stained using 3,3′-diaminobenzidine (DAB), followed by hematoxylin staining (Solarbio, Beijing, China). The stained sections were examined under a light microscopically (Leica, Bensheim, Germany). For IHC quantification, one representative section from each animal was analyzed. Five microscopic fields were randomly selected from each section and quantified using ImageJ 1.48v (NIH, Bethesda, MD, USA). The positive staining area was expressed as the percentage of the total tissue area. All image acquisition and quantitative analyses were performed by an investigator blinded to the treatment groups.

### 2.4. Cell Viability and Inflammation Determination

HGFs were purchased from Innoprot (Bilbao, Bizkaia, Spain) and cultured in human fibroblast expansion medium (Gibco, Grand Island, NY, USA) in an atmosphere of 5% CO_2_ at 37 °C. Cell viability was determine using MTT assay (Sigma, Darmstadt, Germany) after treatment with various concentrations of LM for 24 h.

HGFs were pretreated with LM at 0.5, 1, and 2 (μg/mL) for 3 h, followed by stimulation with PG-LPS at 1 μg/mL (InvivoGen, Hong Kong, China) for 24 h. The levels of PGE2, IL-6, and TNF-α were quantified using ELISA kits (Abcam). Moreover, NO production was detected indirectly by quantifying nitrite (NO_2_^−^), a stable oxidation product of NO, using the Griess Reagent System (Promega, Madison, WI, USA). All experiments were independently repeated three times.

### 2.5. qRT-PCR

Total RNA was isolated using the TaKaRa MiniBEST RNA Extraction Kit (TaKaRa, Tokyo, Japan) according to the manufacturer’s instructions. qRT-PCR was performed using a qTOWER3 real-time PCR system (Analytik Jena, Jena, Germany). Gene expression was analyzed by the 2^−ΔΔCT^ method, with the primer details provided in Table 1.

### 2.6. Western Blot Analysis

Cellular proteins were isolated using RIPA buffer (Biosolution, Seoul, Republic of Korea) and placed on ice for 30 min. Next, the protein mixtures were centrifuged at 12,000 rpm for 10 min at 4 °C, and the supernatants were collected. The protein concentrations were determined using a BCA assay kit (Thermo Scientific, Waltham, MA, USA). Equal amounts of protein were mixed with 5× loading buffer (Biosesang, Seoul, Republic of Korea), heated at 100 °C for 10 min, separated by 10% SDS-PAGE gels, and transferred onto PVDF membranes (Millipore, Burlington, MA, USA).

The membranes were blocked with 3% BSA (Thermo Scientific) dissolved in TBST (Biosesang) for 1 h, then incubated overnight at 4 °C with primary antibodies (Abcam) against iNOS (ab178945, 1:1000), COX-2 (ab179800, 1:1000), p65 (ab16502, 1:1000), pp65 (ab76302, 1:1000), STAT3 (ab68153, 1:1000), and pSTAT3 (ab32143, 1:1000). After three washes with TBST, the membranes were incubated with HRP-conjugated secondary antibodies (Abcam) for 2 h at 37 °C. Finally, immunoreactive bands were visualized using Clarity Western ECL Substrate (Bio-Rad, Hercules, CA, USA) and developed using CL-XPosure Film (Agfa, Mortsel, Belgium). Band intensities were measured with ImageJ 1.48v (NIH). The levels of phosphorylated proteins were normalized to their corresponding total protein levels, while total protein expression was normalized to GAPDH. All experiments were performed independently in triplicate.

### 2.7. Statistical Analysis

Data were analyzed with GraphPad Prism 9.5.1 and expressed as mean ± SD. Normality was evaluated using the Shapiro–Wilk test. Otherwise, the data were analyzed using non-parametric methods (Kruskal–Wallis test) instead of parametric tests. Group differences were examined by one-way ANOVA followed by Tukey’s post hoc test, with *p* < 0.05 considered statistically significant.

## 3. Results

### 3.1. LM Reduced NO and PGE2 Production in HGFs

**Figure 1:**
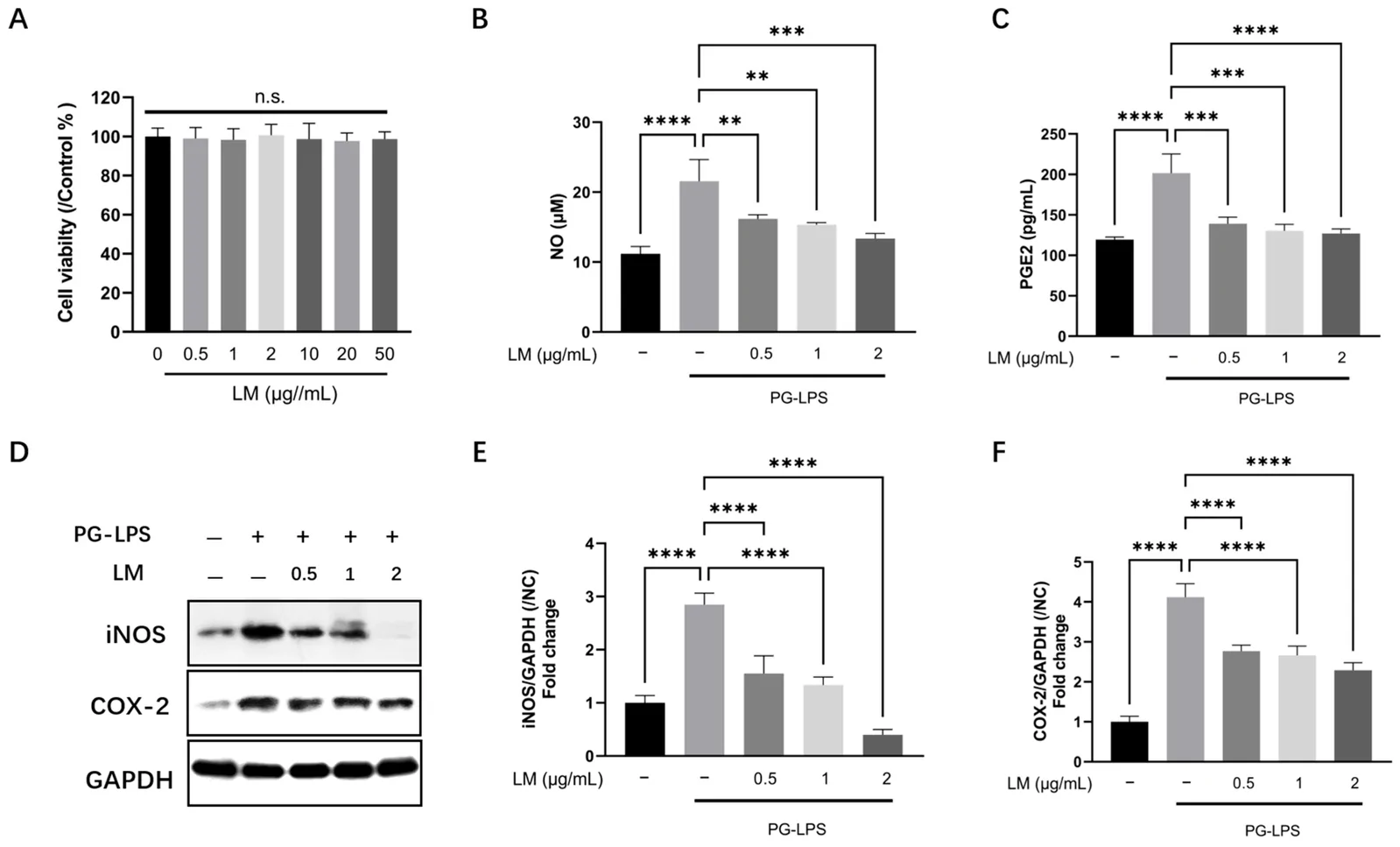
LM reduced NO and PGE2 production in HGFs. (**A**) HGFs were incubated with various concentration of LM (0–50 μg/mL) for 24 h. Cell viability was assessed using the MTT assay. HGFs were pretreated with LM at 0.5, 1, 2 μg/mL for 3 h and stimulated with *P. gingivalis* lipopolysaccharide (PG-LPS, 1 μg/mL) for 24 h. The (**B**) NO production was assessed by quantifying nitrite levels using the Griess Reagent System. (**C**) PGE2 was measured using an ELISA kit. (**D**–**F**) Evaluation of the protein expression levels of iNOS and COX-2 using Western blot. The expression levels of proteins were normalized to GAPDH. Data are expressed as mean ± SD (*n* = 3). Differences between groups were evaluated using one-way ANOVA with Tukey’s post hoc test applied. ** *p* < 0.01, *** *p* < 0.001, **** *p* < 0.0001. n.s., no significant difference.

To assess the cytotoxicity of LM, HGFs were treated with LM (0–50 μg/mL) for 24 h. LM exerted no significant cytotoxicity, as evidenced by unchanged cell viability across all tested concentrations (Figure 1A).

We next examined the effects of LM on the inflammatory response in HGFs. PG-LPS stimulation significantly increased the production of NO and PGE2 (Figure 1B,C). However, treatment with LM at 0.5, 1, and 2 μg/mL markedly reduced the levels of both mediators. Consistent with these findings, PG-LPS significantly upregulated iNOS and COX-2 expression, whereas LM treatment effectively suppressed the expression of both proteins (Figure 1D–F).

### 3.2. LM Attenuated Inflammatory Response in HGFs

**Figure 2:**
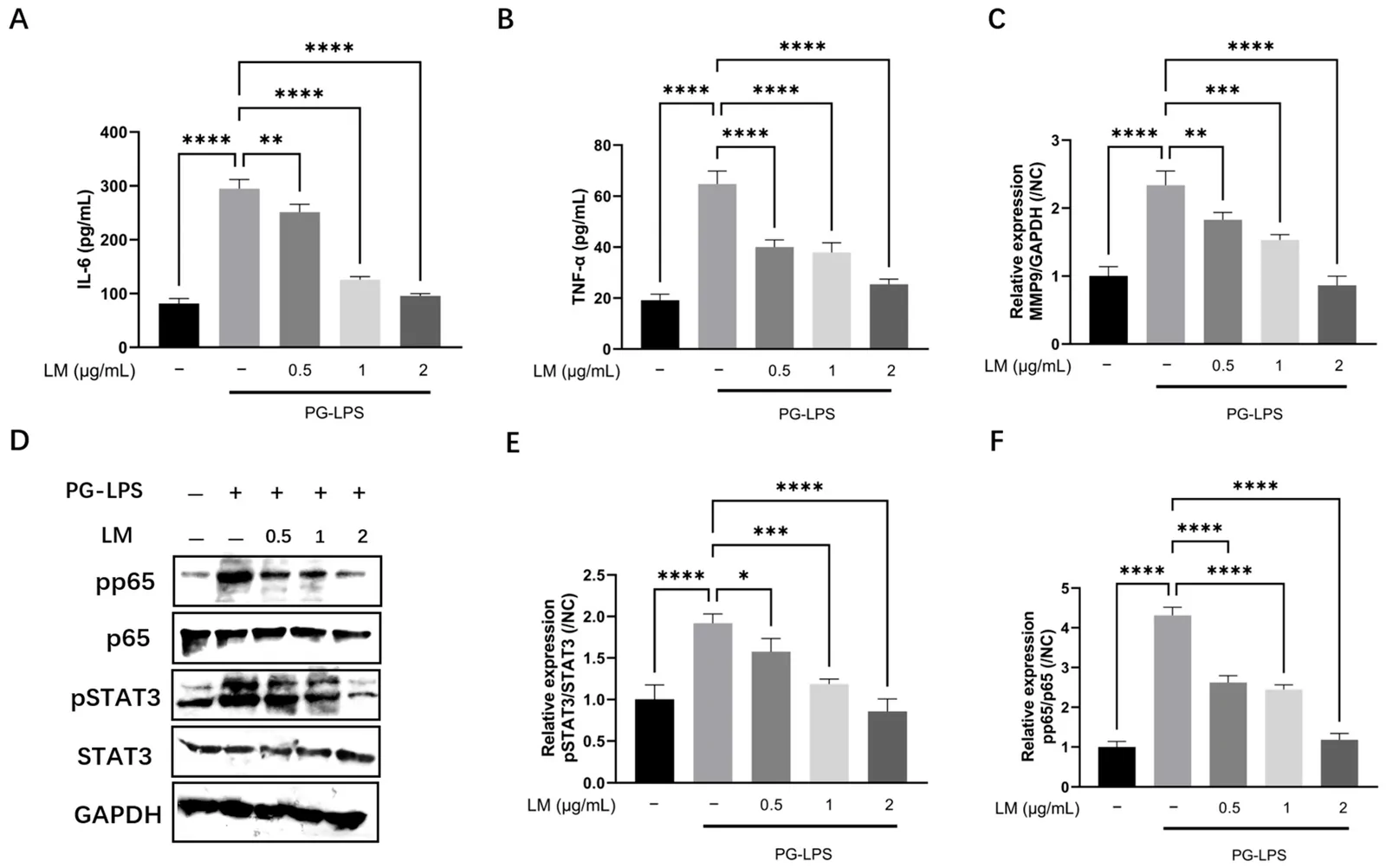
LM inhibited inflammation in HGFs. HGFs were treated with LM at 0.5, 1, 2 μg/mL for 3 h, followed by stimulation with PG-LPS (1 μg/mL) for an additional 24 h. The levels of (**A**) IL-6, and (**B**) TNF-α were quantified using ELISA kits. (**C**) The gene expression of MMP9 was determined using qRT-PCR. (**D**–**F**) Western blot was applied to determine the expression of pSTAT3 and pp65. The expression levels of phosphorylated proteins were normalized to their corresponding total protein levels, while total protein expression was normalized to GAPDH. Data are expressed as mean ± SD (*n* = 3). Differences between groups were evaluated using one-way ANOVA with Tukey’s post hoc test applied. * *p* < 0.05, ** *p* < 0.01, *** *p* < 0.001, **** *p* < 0.0001.

We next determined the levels of pro-inflammatory cytokines in HGFs culture supernatants. PG-LPS stimulation obviously increased the secretion of IL-6 and TNF-α (Figure 2A,B), whereas LM treatment markedly reduced the levels of both cytokines. Moreover, PG-LPS markedly upregulated MMP9 expression, which was significantly suppressed by LM treatment (Figure 2C).

To further investigate the molecular mechanisms underlying the anti-inflammatory effects of LM, we examined the STAT3 and NF-κB signaling pathways. PG-LPS significantly increased pSTAT3, which was dramatically downregulated with LM treatment (Figure 2D,E). Similarly, PG-LPS exposure enhanced pp65 level, while LM treatment markedly reduced pp65 level (Figure 2D,F).

### 3.3. LM Alleviated Gingival Inflammation and Alveolar Bone Loss in the Ligature-Induced PD Rat Model

**Figure 3:**
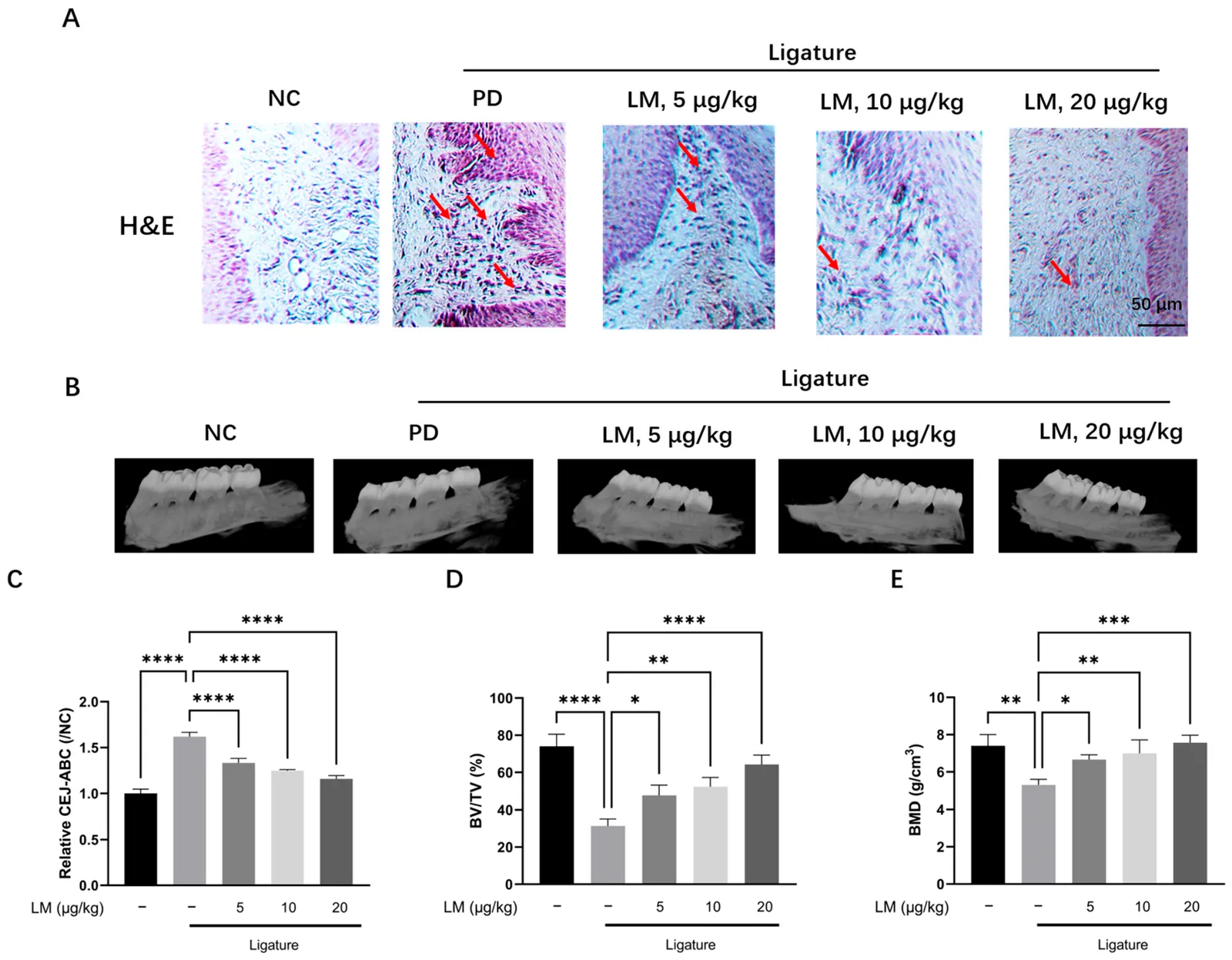
LM reduced gingival inflammation and alveolar bone loss in the ligature-induced PD rats. (**A**) Representative H&E-stained sections of gingival tissue. Scale bar: 50 μm. Red arrows: areas of inflammatory cell infiltration. (**B**) Representative micro-CT images of the maxillary alveolar bone. (**C**) Quantification of alveolar bone loss based on the distance between the cementoenamel junction (CEJ) and the alveolar bone crest (ABC) at the maxillary second molar. (**D**) BV/TV (%) and (**E**) BMD were measured by CATn. Data are expressed as mean ± SD (*n* = 3). Differences between groups were evaluated using one-way ANOVA with Tukey’s post hoc test applied. * *p* < 0.05, ** *p* < 0.01, *** *p* < 0.001, **** *p* < 0.0001.

In the PD rat model, ligature placement resulted in marked inflammatory cell infiltration in the gingival tissues. These histopathological changes were attenuated by LM treatment (Figure 3A).

Micro-CT analysis was performed to evaluate the alveolar bone morphology around the maxillary second molar (Figure 3B). The CEJ–ABC distance in the PD group was greater than that in the NC group, indicating significant alveolar bone loss. Oral administration of LM (5, 10, and 20 μg/kg) significantly reduced the CEJ–ABC distance compared with the PD group (Figure 3C). Consistent with these findings, both BV/TV and BMD were markedly decreased in the PD group, whereas LM treatment restored these parameters toward normal levels (Figure 3D,E).

### 3.4. LM Regulated Gingival Inflammation in the Ligature-Induced PD Rat Model

**Figure 4:**
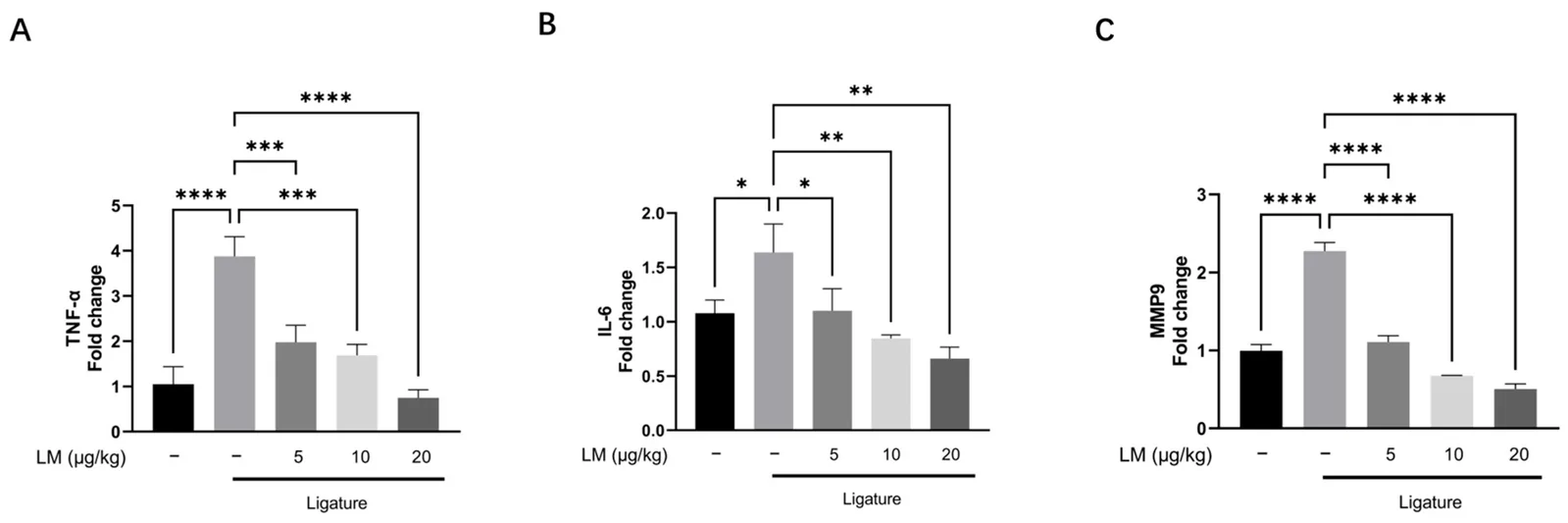
LM reduced inflammatory mediators expression in gingival tissues of ligature-induced PD rats. qRT-PCR was used to determine the mRNA expression levels of (**A**) TNF-α, (**B**) IL-6, and (**C**) MMP9 in the gingival tissues from ligature-induced PD in rats. Data are expressed as mean ± SD (*n* = 5). Differences between groups were evaluated using one-way ANOVA with Tukey’s post hoc test applied. * *p* < 0.05, ** *p* < 0.01, *** *p* < 0.001, **** *p* < 0.0001.

qRT-PCR analysis was performed to evaluate the mRNA expression of TNF-α, IL-6 and MMP9 in the gingival tissues. The mRNA expression levels of TNF-α and IL-6 were significantly higher in the PD group than in the NC group, indicating an enhanced inflammatory response induced by ligature placement (Figure 4A,B). In contrast, LM treatment significantly reduced the expression of both cytokines. Similarly, MMP9 mRNA expression was approximately 2.2-fold higher in the PD group and was significantly decreased after LM treatment (Figure 4C).

### 3.5. LM Attenuated Periodontal Inflammation Associated with Suppression STAT3 Signaling in the Ligature-Induced PD Rat Model

**Figure 5:**
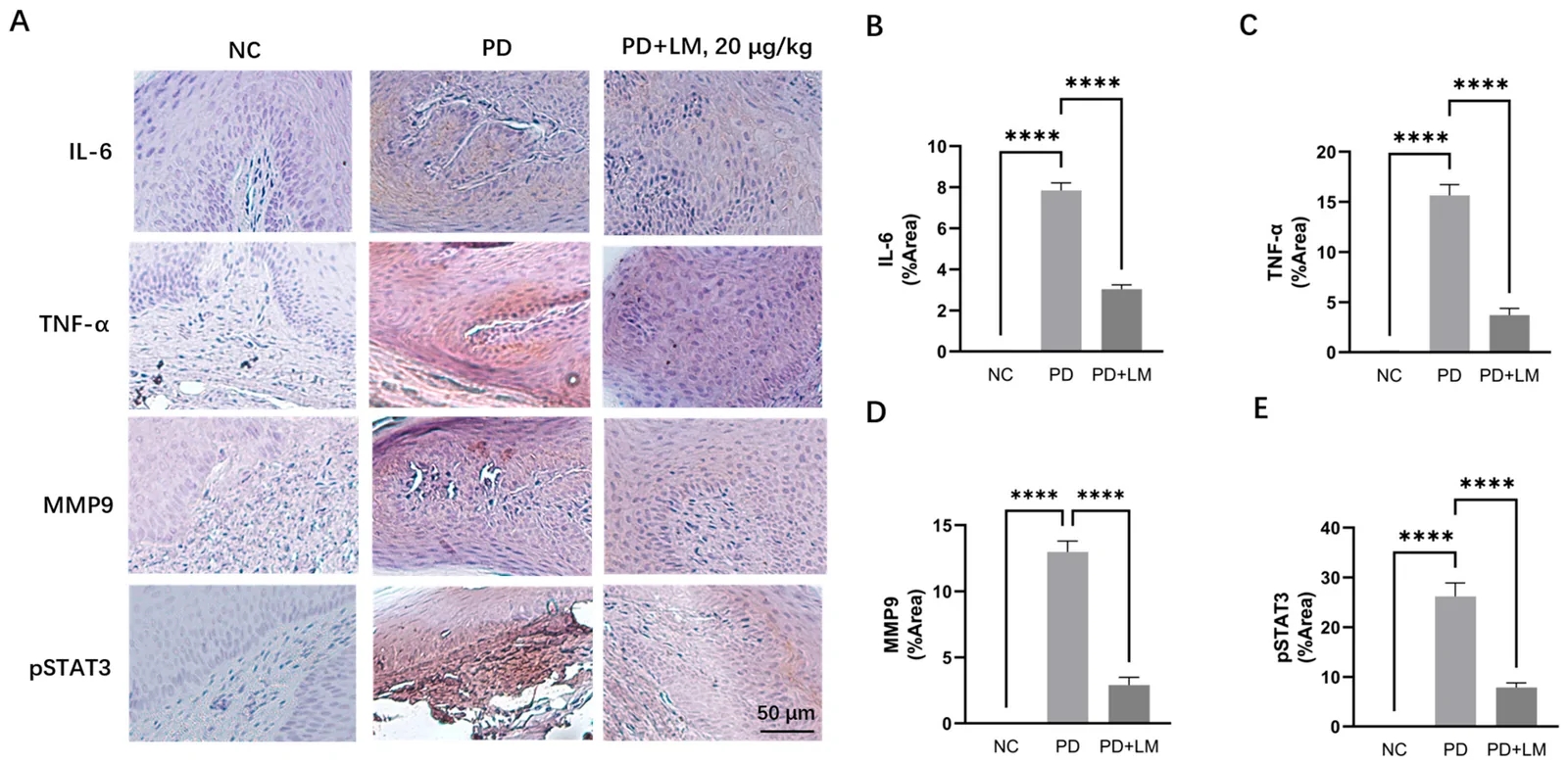
LM suppressed STAT3 activation in the ligature-induced PD rat model. (**A**) Representative immunohistochemical staining images showing IL-6, TNF-α, MMP9, and pSTAT3 expression in gingival tissues. Scale bar: 50 μm. (**B**–**E**) Quantitative analysis of immunohistochemical staining intensity was performed using ImageJ software. Data are expressed as mean ± SD (*n* = 3). Differences between groups were evaluated using one-way ANOVA with Tukey’s post hoc test applied. **** *p* < 0.0001.

Compared with the NC group, IHC analysis revealed significantly increased expression of IL-6, TNF-α, and MMP9 in the gingival tissues of the PD group (Figure 5A–D). Notably, treatment with 20 μg/kg LM markedly reduced the expression of these inflammatory markers. In addition, LM significantly decreased pSTAT3 expression (Figure 5A,E), suggesting that its protective effects against PD are associated with suppression of STAT3 signaling.

## 4. Discussion

PD is initiated and driven by microbial dysbiosis, including periodontal pathogens such as *P. gingivalis*, which activate innate immune responses and induce the production of pro-inflammatory cytokines and chemokines, ultimately leading to the destruction of gingival tissues, periodontal ligaments, and alveolar bone [26,27]. Accumulating evidence highlights the therapeutic potential of SPMs in the treatment of periodontitis [18,19,20]. Previous studies have demonstrated that RvE1 not only suppresses inflammation but also promotes the regeneration of periodontal tissues and alveolar bone in experimental models of PD [19,28,29]. Consistent with previous reports, our results demonstrated that LM exerted potent anti-inflammatory and tissue-protective effects in PG-LPS-stimulated HGFs and a ligature-induced rat model of PD.

Because gingival fibroblasts are the predominant resident cells in the periodontal connective tissue, they play a pivotal role in orchestrating the host inflammatory response following bacterial stimulation. Therefore, targeting inflammatory activation in HGFs represents an important strategy for preserving periodontal tissue homeostasis [12,13,20]. At the cellular level, LM effectively suppressed the production of key inflammatory mediators, including NO and PGE2, by downregulating iNOS and COX-2 expression [30,31]. Given that these mediators contribute to oxidative stress and tissue damage in PD, their inhibition indicates that LM may facilitate the resolution of inflammation during the early phase of PD. NF-κB is a central regulator of inflammation in PD, and controls the expression of pro-inflammatory cytokines, inducible enzymes, and MMPs [26,32,33]. Therefore, inhibition of NF-κB signaling represents a key mechanism underlying the anti-inflammatory effects of LM. In this study, PG-LPS activated the NF-κB signaling pathway, whereas LM treatment markedly suppressed its activation. Consistently, LM also reduced the production of IL-6, TNF-α, and MMP9, further confirming its anti-inflammatory activity. At the tissue level, histopathological examination revealed marked inflammatory cell infiltration in the gingival tissues of the PD group, whereas LM treatment effectively reduced inflammatory cell infiltration and attenuated gingival inflammation. Furthermore, IHC analysis demonstrated that LM markedly decreased the elevated expression of IL-6, TNF-α, and MMP9 induced by ligature placement. These findings indicate that LM effectively attenuate periodontal inflammation.

Persistent inflammation disrupts the balance between bone formation and bone resorption, leading to excessive osteoclast activity and alveolar bone destruction. Therefore, attenuation of periodontal inflammation is associated with preservation of alveolar bone integrity [24,27]. Consistent with the histopathological findings, LM significantly attenuated alveolar bone loss, reducing the CEJ–ABC distance while improving both BV/TV and BMD. These results suggest that LM effectively preserve alveolar bone architecture and mineralization during periodontal inflammation.

In addition to NF-κB, STAT3 is a key signaling mediator that integrates inflammatory signals downstream of cytokines such as IL-6 [34,35,36]. Sustained STAT3 activation amplifies inflammatory responses by promoting the expression of pro-inflammatory cytokines [37,38]. Furthermore, activated STAT3 promotes receptor activator of nuclear factor-κB ligand (RANKL) expression and shifts the RANKL/osteoprotegerin (OPG) balance, thereby promoting osteoclast differentiation and activation. Collectively, persistent STAT3 activation contributes not only to inflammation but also to alveolar bone resorption, making it an attractive therapeutic target for preventing PD progression [39,40,41]. In our previous study, LM suppressed osteoclast differentiation and downregulated the expression of osteoclast-associated genes, including cathepsin K (CTSK) and tartrate-resistant acid phosphatase (TRAP), in RANKL-stimulated RAW264.7 cells, accompanied by inhibition of STAT3 signaling [23]. Consistent with our previous findings, the present study further demonstrated that LM suppressed STAT3 phosphorylation in both PG-LPS-stimulated HGFs and gingival tissues from ligature-induced PD rats. Collectively, these results suggest that suppression of STAT3 signaling may contribute, at least in part, to the protective effects of LM against periodontal inflammation and alveolar bone loss.

## 5. Conclusions

Taken together, these findings suggest that LM alleviated periodontal inflammation and alveolar bone loss, and that these protective effects were associated with suppression of the NF-κB and STAT3 signaling pathways. The present findings indicate that suppression of STAT3 signaling may contribute to the protective effects of LM; however, further studies directly evaluating osteoclastogenesis and bone remodeling pathways are required to establish the underlying mechanisms.

In addition, the biological effects observed in this study cannot be attributed to any individual lipid mediator and are likely the result of the combined actions of the LM mixture. Therefore, further studies evaluating the effects of the individual lipid mediators, together with appropriate comparator controls, will be essential for defining their respective roles in periodontitis. Moreover, additional studies investigating the pharmacokinetic properties, tissue distribution, and safety profile of orally administered LM are required to determine its clinical potential. Because only male animals were included in the present study, future investigations involving both male and female animals will be important to further validate our findings. Finally, studies employing bacteria-induced periodontitis models or clinically relevant polymicrobial infection models will be valuable for further confirming the therapeutic efficacy and translational potential of LM.

## Figures and Tables

**Table 1:** Primers.

Genes	Sequences (5′→3′)
Rat IL-6	Forward: CAATGAGGAGACTTGCCTGG
	Reverse: TGGGTCAGGGGTGGTTATTG
Rat TNF-α	Forward: CCCATGTTGTAGCAAACCCTC
	Reverse: TATCTCTCAGCTCCACGCCA
Rat MMP9	Forward: CGCTGGGCTTAGATCATTCT
	Reverse: GAGCCACGACCATACAGATG
Rat β-Actin	Forward: TCCTCCTGAGCGCAAGTACTCT
	Reverse: GCTCAGTAACAGTCCGCCTAGAA
Human MMP9	Forward: GCCACTACTGTGCCTTTGAGTC
	Reverse: CCCTCAGAGAATCGCCAGTACT
Human β-Actin	Forward: CACCATTGGCAATGAGCGGTTC
	Reverse: AGGTCTTTGCGGATGTCCACGT

## Data Availability

The data presented in this study are available from the corresponding author upon reasonable request.

## Supplementary Materials

The following supporting information can be downloaded at: https://www.mdpi.com/article/10.3390/nu18162659/s1, Figure S1: Preparation and characterization of the LM mixture.
