# Supplementary material for: Lipid Mediators Derived from Docosahexaenoic Acid Alleviate Periodontal Inflammation and Alveolar Bone Loss in Periodontitis Associated with Suppression of the NF-κB and STAT3 Signaling Pathways
Source: Nutrients. 2026 Aug 14;18(16):2659. doi: 10.3390/nu18162659 (PMC13517078; doi:10.3390/nu18162659)
Supplement: Supplementary file 1 [file nutrients-18-02659-s001.zip › nutrients-4358702-supplementary.pdf]

Preparation and characterization of the LM mixture

The LM mixture was enzymatically generated from docosahexaenoic acid (DHA) using soybean lipoxygenase derived from soy flour. Briefly, 100 μM DHA was incubated with 25 μL of soy flour extract in 10 mL phosphate buffer (50 mM, pH 7.0) at room temperature for 15 min. Under these reaction conditions, three major lipid mediators were generated. The reaction products were separated by normal-phase high-performance liquid chromatography (NP-HPLC), and the lipid mediator fractions were subsequently characterized by liquid chromatography–mass spectrometry (LC-MS) and liquid chromatography–tandem mass spectrometry (LC-MS/MS). The LM mixture consisted of 17S-hydroxydocosahexaenoic acid (17S-HDHA), resolvin D5 (RvD5), and protectin DX (PDX) at a relative ratio of 3:47:50. Representative chromatograms and mass spectra are provided Supplementary Figure S1.

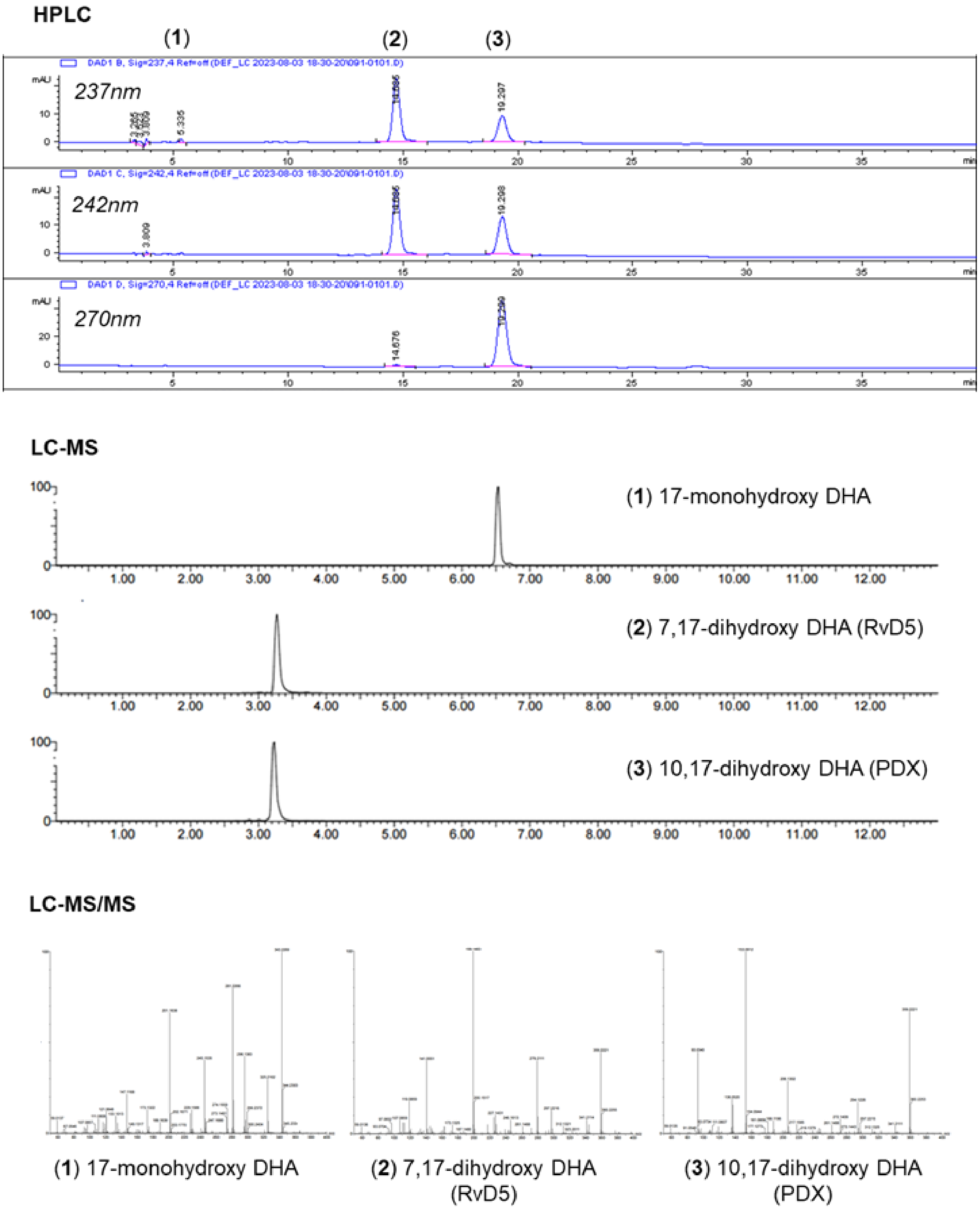

Supplementary Figure S1**.** Preparation and characterization of the LM mixture.
